# Exploring Facilitators and Barriers to Delayed Antibiotic Prescribing in Rural Northwest China: A Qualitative Study Using the Theoretical Domains Framework and Behavior Change Wheel

**DOI:** 10.3390/antibiotics12121741

**Published:** 2023-12-15

**Authors:** Haishaerjiang Wushouer, Weihsin Ko, Kexin Du, Wanmeng Zhang, Lin Hu, Junxuan Yu, Kairui Zhang, Luwen Shi, Xiaodong Guan

**Affiliations:** 1 Department of Pharmacy Administration and Clinical Pharmacy, School of Pharmaceutical Sciences, Peking University, Beijing 100191, China; kaiser@pku.edu.cn (H.W.); 2211110175@stu.pku.edu.cn (W.K.); 2011110135@stu.pku.edu.cn (K.D.); 2111210065@bjmu.edu.cn (W.Z.); michelle_hlin@pku.edu.cn (L.H.); 2211210069@stu.pku.edu.cn (J.Y.); zhangkarry@stu.pku.edu.cn (K.Z.); 2 International Research Center for Medicinal Administration, Peking University, Beijing 100191, China

**Keywords:** delayed antibiotic prescribing, antibiotic use, Theoretical Domains Framework, Behavior Change Wheel

## Abstract

Background: Antimicrobial resistance, exacerbated by antibiotic misuse, poses a global threat. Though delayed antibiotic prescribing (DAP) can mitigate antibiotic overuse, its adoption in developing nations, such as China, is limited. This study probed barriers and facilitators to DAP in Xinjiang, characterized by extensive rural landscapes and primary care institutions (PCIs). Methods: Adopting a qualitative methodology, we conducted key informant interviews with thirty participants across six county hospitals in Xinjiang using VooV Meeting. Employing a two-stage sampling method targeting economically diverse areas, our interviews spanned physicians, pharmacists, patients, and caregivers. We organized the data according to the Theoretical Domains Framework (TDF) and the Behavior Change Wheel (BCW), spotlighting behavioral and policy elements impacting DAP. Results: Our research included thirty interviewees. Twelve physicians contemplated delayed prescriptions, while five adult patients and six caregivers encountered recommendations for delayed antibiotic prescriptions. Six patients sought pharmacists’ advice on antibiotic necessity. Prominent TDF domains were memory, attention, and beliefs about consequences. Critical intervention functions included education and environmental restructuring, while vital policy categories encompassed communication/marketing and guidelines. Conclusions: Countering antibiotic misuse and resistance in China necessitates overcoming barriers through strategic resource distribution, comprehensive education, rigorous training, and consistent monitoring, thereby promoting DAP adoption. The adoption of DAP in rural healthcare settings in China has the potential to significantly reduce antibiotic misuse, thereby mitigating the global threat of antimicrobial resistance.

## 1. Introduction

Antimicrobial resistance (AMR) is a daunting public health challenge that countries worldwide are currently grappling with. As reported by the World Health Organization, AMR was associated with approximately 4.95 million deaths in 2019, and the economic burden is projected to soar from 300 billion USD to over 1 trillion USD by 2050 [1,2]. This calls for urgent interventions, including the judicious use of antibiotics, especially the “Watch” and “Reserve” categories, while promoting “Access” antibiotics to mitigate AMR’s impact [3].

In response, many high-income countries have implemented measures to enhance collaborative decision-making between physicians and patients. Delayed antibiotic prescribing (DAP) is one of the most implemented interventions and has been gaining traction among health management researchers and been incorporated into some countries’ guidelines [4,5]. According to the National Institute for Health and Clinical Excellence, DAP involves a physician providing a prescription during a consultation but asking the patient to delay its use to see if symptoms resolve first. A Cochrane systematic review reported no difference in symptoms or complications between adults and children with delayed antibiotic prescribing, immediate antibiotic prescription, and no antibiotic prescription. In addition, the study revealed a significant reduction in antibiotic usage associated with delayed antibiotic prescribing compared to immediate antibiotic prescription. In particular, the revisitation rates were similarly low in the delayed antibiotic prescribing group, as compared to the immediate antibiotic prescription group [6,7].

However, DAP is scarcely employed in developing countries. Notably, in China, despite nearly 20 years of antimicrobial stewardship, primary care institutions (PCIs) still face challenges in terms of rational use of antibiotics. PCIs provide basic medical services for most of the population. According to data from the National Health and Family Planning Commission of China, there were 8.47 billion medical visits nationwide in 2018, of which 4.25 billion (50.18%) were in the PCIs [8]. Notably, between 1 October 2014 and 30 April 2018, a significant 51.4% of all ambulatory care antibiotic prescriptions in China were considered inappropriate [9]. A previous study pointed out that 42% (221/526) of the cases in rural China’s PCIs were inappropriately prescribed antibiotics [10]. China’s “National Action Plan to Contain Bacterial Resistance (2016–2020)” marked significant progress in antibiotic management, resulting in notable reductions in antibiotic use [11]. However, challenges of resistance in common microbes persist. The subsequent “National Action Plan to Curbing Microbial Drug Resistance (2022–2025)” seeks to extend these efforts, emphasizing prevention and comprehensive strategies [12]. Despite outlining tasks, such as enhancing public health education and strengthening training, detailed procedural planning for achieving a 60% correct usage rate of antimicrobial drugs remains a gap. This emphasizes the need to explore alternative strategies, such as DAP.

This situation in China reflects a global challenge, as many countries struggle with similar issues of antibiotic overuse and resistance. Currently, DAP has not been introduced in China. DAP could be a potential intervention to adopt for China’s PCIs to promote rational use of antibiotics; however, little is known about the feasibility of the potential implementation of DAP in China’s PCIs. Therefore, we aim to investigate the potential barrier and facilitator to the adoption of DAP in the rural regions of western China. The exploration of DAP’s feasibility and implementation in rural China could provide valuable insights applicable to other developing countries facing similar challenges.

## 2. Results

A total of 30 interviewees were included in the study. Among them, 12 physicians reported considering the use of delayed prescriptions as a strategy. Additionally, five adult patients and six caregivers received advice to fill a DAP, while six patients consulted pharmacists to determine the necessity of taking antibiotics.

**Figure 1:**
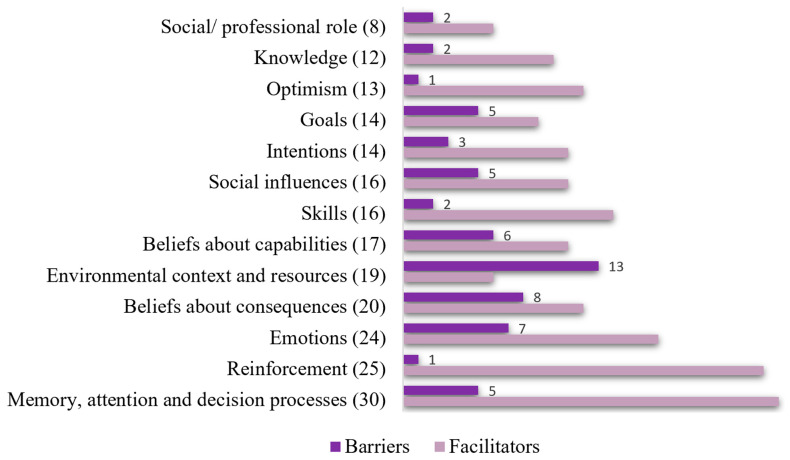
A histogram of the relationship between barriers and facilitators in each domain of the Theoretical Domains Framework. The numbers indicate the count of identified barriers and facilitators within each domain, such as Memory, Attention, and Decision Processes (*n* = 30), accounting for 13.1% of the factors, indicating this as one of the most frequently identified domains. The figure also notes the overall percentages for each domain and mentions any domains not observed in the study, such as Behavioral Regulation.

Figure 1 depicts the relationship between barriers and facilitators within each TDF domain. Facilitators accounted for 73.7% of all factors, while barriers constituted the remaining factors. The most commonly coded TDF domains, representing approximately 79.5% of total barriers and facilitators, were memory, attention, and decision processes (*n* = 30; 13.1%), reinforcement (*n* = 25; 10.9%), emotions (*n* = 24; 10.5%), beliefs about consequences (*n* = 20; 8.7%), environmental context and resources (*n* = 19; 8.3%), beliefs about capabilities (*n* = 17; 7.4%), skills (*n* = 16; 7%), social influences (*n* = 16; 7%), and intentions (*n* = 14; 6.1%). It is noted that no results pertaining to behavioral regulation were observed, as the delayed antibiotic prescription had not been implemented at the time of the study.

### 2.1. Knowledge about DAP

It was found that both physicians and pharmacists exhibited knowledge and understanding of DAP, appropriate antibiotic use, and symptomatic management. They recognized the significance of avoiding unnecessary antibiotic prescriptions and acknowledged the potential benefits of implementing delayed prescribing strategies. Moreover, they demonstrated familiarity with symptom management and the appropriate utilization of antibiotics for various conditions.

Notably, N2’s statement highlighted the potential advantages of employing DAP to promote responsible antibiotic use. By carefully evaluating patients’ symptoms and the likelihood of bacterial infection, healthcare professionals can effectively reduce unnecessary antibiotic use while still ensuring appropriate treatment for those in need. Additionally, the utilization of DAP can help mitigate the risks associated with antibiotic use, including the development of antibiotic resistance and other adverse health effects.

The demonstrated knowledge and understanding among physicians and pharmacists indicate their readiness to embrace DAP and make informed decisions regarding antibiotic prescriptions. This signifies a positive attitude toward implementing evidence-based practices to optimize antibiotic use and enhance patient outcomes.

“Antibiotics should not be prescribed when they (patients) are not needed.”

—N2

“I had read the research done by the University of Southampton in the UK, and they thought that the DAP would not exacerbate patient’s symptoms and could potentially improve their prognosis and reduce the risk of recurrent disease.”

—N211

Among the surveyed pharmacists, five out of six reported observing physicians utilizing DAP in their practice. N23’s statement underscored the importance of tailoring antibiotic use to the specific needs of individual patients and their conditions. By carefully assessing patients’ symptoms and determining the likely cause of respiratory distress, healthcare professionals can make informed decisions about whether and when to prescribe antibiotics. In certain situations, as described by N23, DAP can effectively reduce unnecessary antibiotic use while still ensuring that effective treatment is provided when necessary.

“When I practiced in the respiratory department, antibiotics were generally delayed prescribed if a patient had viral pneumonia or bronchial asthma without other infectious symptoms. In addition, in mild cases of COPD, antibiotics are not used at the beginning of treatment.”

—N23

One adult patient exhibited understanding of appropriate antibiotic use, as evidenced by the statement provided by N19. This observation suggests a growing awareness among patients regarding the potential risks associated with unnecessary antibiotic use. Patients are actively seeking guidance to ensure the judicious utilization of these medications.

“We often hear about this on the news or read about it online, so try to use antibiotics sparingly.”

—N219

### 2.2. Skills

All physicians and the majority of pharmacists (four out of six) who participated in the interviews demonstrated proficient communication skills necessary for effective implementation of the DAP with their patients. Notably, these healthcare professionals recognized the significance of adopting a caring and responsible approach when engaging in discussions about antibiotic use with their patients.

“Give him (a patient) a detailed explanation (about why DAP is implemented).”

—N21

“We should be a caring person…Follow up well and give patients a sense of trust (be responsible).”

—N26

“The patient says, ‘Why didn’t the physician prescribe antibiotics for me? I said I had a sore throat and was uncomfortable, but the physician didn’t prescribe medication for me.’ In such cases, we will explain to them.”

—N28

However, it is worth noting that two out of the six pharmacists interviewed expressed the perspective that it is primarily the responsibility of physicians to explain to patients why it is necessary to implement the DAP.

“Communicate with the physician and let the physician explain to the patient again.”

—N23

“If they (patient) don’t ask me about it (delayed prescribed), we usually don’t explain it.”

—N23

Among the 12 physicians and pharmacists interviewed, it is noteworthy that 5 physicians and 1 pharmacist expressed a strong endorsement of the correct concept of antibiotic use based on their medical knowledge.

“We must adopt scientific approaches and theories to explain (to patients) why they are not used.”

—N212

“Inform patients that if they use higher-grade antibiotics, they will develop bacterial resistance and become insensitive to antibiotics and that fewer antibiotics will be available in the future.”

—N23

Lastly, it is worth noting that a physician and a pharmacist shared a mutual agreement regarding the importance of possessing knowledge about antibiotic use when utilizing or explaining DAP.

“It (whether to use an antibiotic) requires our clinical experiences, a deep understanding of respiratory diseases, and a precise grasp of guidelines.”

—N212

“Relatives and friends will consult me by phone, and I will recommend which medicine is suitable for him.”

—N28

### 2.3. Memory, Attention, and Decision Processes

All physicians who participated in the study relied on a patient’s clinical symptoms and the results of auxiliary examinations as the basis for their decision to prescribe antibiotics or utilize DAP. They highlighted the significance of integrating scientific knowledge and drawing from their own clinical experiences when making these crucial decisions.

“The patient has no sore throat or fever, no pulmonary rales, and the examination of the image is not consistent with bacterial infection; antibiotics should not be prescribed in this situation.”

—N2

“We still decide (whether to prescribe an antibiotic or not) by checking the infection indicators.”

—N22

They further acknowledged that the decision to prescribe antibiotics was contingent upon several factors, including the severity and type of the disease.

“It (DAP) depends on the disease. Patients with COPD who have had a good therapeutic effect on previous macrolides may be given antibiotics by the patient’s will during the acute episode.”

—N7

In certain situations, physicians acknowledged the practical challenge of delayed pathogenic test results and made the decision to prescribe antibiotics empirically.

“We will administer the medication empirically until the results of his pathogenic tests are available.”

—N11

In contrast to physicians, pharmacists in the study reported limited involvement in the decision-making process for DAP. Two out of six pharmacists expressed that their input or opinion regarding DAP was not considered useful in these situations, and they deferred to the physician’s judgment.

“The physician determines the severity of the patient’s symptoms through questioning and thus decides whether to prescribe antibiotics.”

—N23

“…our opinion is not useful (for patients with delayed prescriptions).”

—N218

When patients sought guidance from pharmacists regarding delayed antibiotic prescriptions, the pharmacists in the study responded by providing scientific explanations to justify why antibiotics might not be necessary based on the patient’s symptoms and examination results.

“We will tell him that acute upper respiratory tract infection symptoms do not appear immediately, most of which are caused by the virus (scientific knowledge). We advise him (the patient) to drink more water, rest, and observation. Antibiotics should be taken after symptoms like fever and infection become apparent.”

—N28

Overall, the study findings revealed that 8 out of 12 patients and caregivers demonstrated a proactive approach to antibiotic use, either by following the advice of their physicians and delaying the initiation of antibiotics based on clinical symptoms or by making informed decisions based on their own experiences.

“I will go to the hospital for a blood test first, and then I will take it (antibiotic) if the physician tells me to take it or not if he doesn’t.”

—N25

“I will observe (symptoms) for a couple of days and take medication which treats the cold.”

—N230

### 2.4. Social/Professional Role and Identity

Among the 12 patients and caregivers interviewed, it was observed that while 2 individuals expressed satisfaction with their current level of communication with their physicians, the majority (10) of participants identified a need for improved communication with their healthcare providers. Among the 10 individuals who desired better communication, 4 specifically mentioned the need for additional support from pharmacists and nurses. These participants believed that these healthcare professionals could provide more detailed instructions on medication management and offer more frequent check-ins, which would contribute to better understanding and adherence to prescribed treatments. Conversely, two participants expressed that they did not feel the need for further assistance beyond what their physicians provided.

“I don’t think this (help from healthcare professionals besides the physician) is necessary.”

—N24

“I usually don’t need to ask any more (questions about delayed prescriptions) if I follow those instructions to take medicine.”

—N25

### 2.5. Beliefs about Capabilities

Beliefs about healthcare professionals’ capabilities encompass patients’ trust in the expertise of physicians and pharmacists. Among the 12 physicians interviewed, 3 expressed the belief that patients would follow their recommendations because they have trust in their professional expertise.

“Generally, most patients can accept our recommendations.”

—N2, N7, N12

However, among the six pediatricians interviewed, three expressed the viewpoint that some parents do not have sufficient trust in physicians and pharmacists. Consequently, these parents may rely on alternative sources of information or their own judgment when making decisions about whether to delay taking antibiotics.

“Some parents not only listen to the physician but also listen to the judgment of friends, neighbors or themselves.”

—N211

According to one pediatrician, providing a clear and detailed explanation can significantly increase the acceptance rate of recommendations. They reported that as many as 70% to 90% of caregivers would accept the physician’s advice when it is accompanied by a thorough and understandable explanation. Among the 12 patients and caregivers interviewed, 6 individuals reported following the physician’s advice, citing their trust in the physician’s professional expertise as the primary reason.

“I think the physician has his expertise, and I will usually listen to his advice.”

—N25

Among the six pharmacists interviewed, two expressed the belief that most patients would not readily accept individual recommendations from pharmacists. On the other hand, four pharmacists held the perspective that patients could potentially accept their advice after it was thoroughly explained to them.

“Most patients will not accept individual recommendations from pharmacists.”

—N23

“Patients are also relatively receptive to the suggestion of DAP after being explained.”

—N213, N28

Among the 12 patients and caregivers interviewed, 3 individuals explicitly expressed trust in their physician’s expertise and consequently accepted their suggestions.

“After all, physicians must have professional skills, so we must trust them.”

—N29

### 2.6. Optimism

The study revealed that all physicians and the majority of pharmacists (five out of six) exhibited confidence in the effectiveness of the DAP for promoting rational antibiotic use. These healthcare professionals acknowledged the value of DAP in minimizing unnecessary antibiotic prescriptions and reducing the risk of antimicrobial resistance.

“Theoretically, DAP should promote rational antibiotic use, and we are very supportive.”

—N2

### 2.7. Beliefs about Consequences

The study findings revealed a considerable variation in beliefs about the consequences of using DAP among healthcare professionals and patients. Among the 12 physicians interviewed, only 3 expressed confidence in the ability of DAP to improve antibiotic use and reduce bacterial resistance. They recognized the potential benefits of DAP in minimizing unnecessary antibiotic prescriptions and mitigating the risks associated with antibiotic resistance. In contrast, 5 out of 12 physicians expressed concerns about the use of DAP. They raised apprehensions that delayed treatment resulting from DAP could potentially lead to worsened patient outcomes. These healthcare professionals may have reservations about the feasibility and effectiveness of implementing the DAP approach in their clinical practice.

“I don’t take antibiotics if it’s a common cold, because I’m afraid of developing antibiotic resistance.”

—N24

“If the only purpose is to implement the DAP…it will only cause the infection of this child to worsen…Originally, choosing antibiotics like second and third generation can control the infection, but because of the delayed treatment, we will need to select stronger antibiotics.”

—N21

### 2.8. Intentions

Among the physicians interviewed, there was a diversity of opinions regarding patient receptiveness to advice on the DAP. Five out of twelve physicians expressed confidence that patients would be receptive to receiving advice and information about DAP. They believed that patients would be open to understanding the rationale behind delayed prescribing and the potential benefits it offers.

“They generally cooperate well with my diagnosis and treatment.”

—N212

On the other hand, two physicians held a contrasting view and expressed skepticism about patient receptiveness to DAP advice. These physicians may have perceived a potential resistance from patients in accepting the concept of delayed prescribing or may have encountered challenges in effectively communicating the principles of DAP to their patients.

“Among 50% of the children, only one-third of the parents are willing to accept.”

—N26

Among the pharmacists interviewed, two out of six took an active role in explaining DAP to patients. They proactively provided information about the approach, its purpose, and the importance of judicious antibiotic use.

“I will proactively explain the risk of antibiotic resistance to patients.”

—N23

In contrast, one pharmacist adopted a more passive approach and only explained DAP when specifically asked by patients.

“If patients ask, I will explain it to them. If they don’t ask me, I usually won’t explain.”

—N23

### 2.9. Goals

The estimation of the proportion of patients suitable for DAP varied among the healthcare professionals interviewed. Eight out of twelve physicians and one pharmacist believed that more than 50% of patients could be considered suitable for DAP.

“At least 50 to 60 percent of outpatients do not need antibiotics.”

—N216

On the other hand, three out of twelve physicians and two out of six pharmacists expressed the opinion that less than 50% of patients would be suitable for DAP.

“Inpatients are about 30–40%; outpatients are about 50–60% or more.”

—N22

“The proportion is relatively low; I think it is 10–20%.”

—N23

### 2.10. Reinforcement

The findings of the study revealed a consensus among the healthcare professionals regarding several key interventions and strategies to promote rational antibiotic use. Ten physicians and five pharmacists agreed on the importance of clarifying antibiotic use indicators, implementing a reward and punishment system for antibiotic prescriptions, and providing training to physicians to enhance their knowledge and practices related to antibiotic use in hospital management. Furthermore, three physicians and one pharmacist emphasized the need for government management to support hospital and medical personnel in promoting rational antibiotic use.

In addition to hospital and government management, the study also identified the importance of strengthening publicity and education efforts on antibiotic use for both medical personnel and patients. However, it is worth noting that one pharmacist expressed skepticism about the effectiveness of promoting DAP and did not believe there is a viable way to promote it. Additionally, N11 suggested the implementation of a relevant standardized management system to oversee hospital and medical personnel. This system could involve the intervention and collaboration of entities, such as the medical insurance department and the hospital evaluation mechanism.

“There must be a relevant standardized management system to manage, for example, the medical insurance department’s intervention and the hospital’s evaluation mechanism.”

—N211

### 2.11. Emotion

Two out of twelve physicians and two out of six pharmacists believed that patients would be receptive to DAP. However, three physicians and three pharmacists expressed doubts about patients’ acceptance of DAP. It is important to note that it was unclear whether these patients had prior experience with DAP or if they were being introduced to the concept for the first time. Of the eleven patients included in the study, all of them were willing to accept the suggestion of delayed prescription. However, one parent expressed suspicion about DAP and required further explanation from the physician.

“Parents with such aggravation experience may be more cautious or more fearful, afraid that the child will be aggravated after returning home, and then they will take more medicine.”

—N26

“Some people are quite stubborn and may go to the pharmacy to buy medicine by themselves.”

—N217

“I need to hear the doctor’s explanation.”

—N210

### 2.12. Environmental Context and Resources

The study revealed that two physicians and four pharmacists acknowledged the existence of national antibiotic remediation efforts that could support the promotion of DAP. However, the implementation of DAP faces obstacles due to insufficient environmental resources within healthcare settings. Primary hospitals often lack adequate hardware facilities, experience a shortage of healthcare personnel, and have a limited number of professional physicians available. These resource limitations pose challenges to the effective implementation of DAP. Furthermore, inadequate patient publicity and education regarding rational antibiotic use, coupled with a suboptimal healthcare environment, can contribute to patients’ disapproval of hospitals. Another significant aspect identified in the study is the role of government supervision of hospitals. The insufficient oversight of hospitals by the government may impede the full implementation of strategies for rational antibiotic use, including DAP.

“The issue about delaying antibiotics prescription…in fact, may have been quietly done by everyone, but it has not been enforced in words.”

—N22

### 2.13. Social Influences

The study findings indicate a diversity of opinions among healthcare professionals regarding patient acceptance of DAP. Six physicians and four pharmacists expressed the belief that patients, parents, or young individuals with higher education levels and a clear understanding of the disease are more likely to accept DAP. On the other hand, four physicians and two pharmacists held the view that less educated patients, parents, or elderly patients might be less likely to accept DAP. Additionally, the study highlights the challenge of communication between healthcare professionals and patients when a patient’s symptoms do not improve. N7 suggested that in such cases, the effectiveness of communication may be hindered, possibly due to patient frustration or disappointment.

“If the patient’s symptoms improve, it is easy to communicate, but if the symptoms do not improve, it is more difficult to communicate.”

—N27

### 2.14. Behavior Change Wheel of DAP

**Figure 2:**
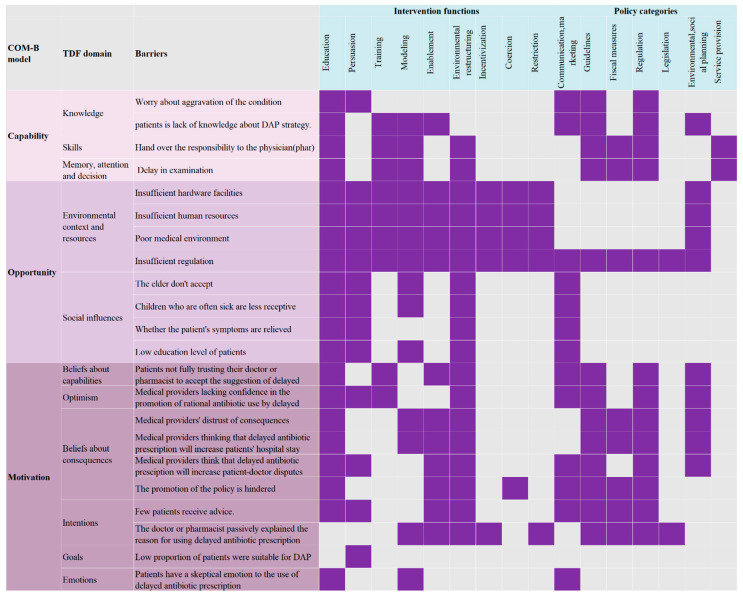
An analysis of potential barriers of adopting delayed antibiotic prescribing using the Behavior Change Wheel. Figure illustrates the alignment of identified barriers within TDF domains with corresponding intervention functions and policy categories of the BCW in the context of DAP implementation. The purple shading denotes the results of this mapping, highlighting the areas of BCW that are most pertinent to addressing the barriers in the implementation process of DAP. Abbreviations: COM-B (Capability, Opportunity, Motivation - Behavior), TDF (Theoretical Domains Framework), and DAP (Delayed Antibiotic Prescribing).

Figure 2 presents the findings of the analysis conducted on the BCW regarding the implementation of DAP. Out of the nine intervention functions examined, education, environmental restructuring, and modeling emerged as the most prominent potential intervention categories that align with the identified barriers. In terms of policy categories, communication/marketing, guidelines, and regulation were identified as the most significant contributors to addressing the identified barriers.

## 3. Discussion

In the realm of delayed antibiotic prescribing (DAP), several significant advantages emerged from our study. Firstly, a profound understanding of DAP by physicians and pharmacists stands as the bedrock for its effective implementation. This foundational knowledge among healthcare professionals ensures that DAP is not only employed but also communicated to patients in a comprehensive and informed manner. Secondly, the noticeable increase in public awareness regarding the risks associated with inappropriate antibiotic use attests to the success of public health initiatives. This growing awareness signifies a shift in public perception, aligning with the goals of responsible antibiotic use and DAP. Furthermore, among healthcare professionals, nearly half of the physicians expressed a preference for DAP. This preference, coupled with discernible awareness among pharmacists and patients, suggests a promising future for DAP’s broad adoption. The trajectory for widespread acceptance is further bolstered by the study’s emphasis on specific cognitive domains within the TDF, such as memory, attention, and decision processes. This focus highlights the pivotal role of consistent reminders and decision-making support in the DAP context. Moreover, central to DAP’s implementation is clinical expertise, with physicians and pharmacists playing pivotal roles. Their collaboration can fine-tune antibiotic prescribing decisions, fostering greater acceptance of DAP. Lastly, the inherent trust relationship between doctors and patients strengthens the receptivity to DAP, laying a solid foundation for its integration into clinical practice.

While DAP presents various advantages, there are anticipated challenges and areas for improvement in its potential implementation. A core challenge is the environmental context and resource constraints, which indirectly impact the confidence and capability of medical professionals in effectively integrating DAP into their practice. Our study identified varied confidence levels among pharmacists, pinpointing an immediate need for specialized training initiatives to enhance their DAP proficiency. Effective clinician–patient communication is paramount, especially in innovative clinical paradigms, such as DAP. The study highlighted a noticeable gap in effectively conveying the intricacies of DAP to patients, emphasizing the need for fortified communication strategies. Current sentiments primarily place the onus of explaining DAP on physicians, revealing deep-seated healthcare hierarchies. This highlights the need for more collaborative educational approaches that involve all healthcare professionals in patient education on DAP. Resources and the environment also indirectly influence patients’ trust in healthcare professionals. Lower satisfaction rates with rural medical services and diminished confidence in healthcare providers can lead to pressured antibiotic prescriptions [13]. Additionally, fluctuating levels of trust, particularly in the context of pharmacy, identify areas requiring improved patient education and refined counseling to build trust in DAP. Other obstacles include low patient education levels, patient age, and inadequate patient awareness. In regions such as China, with prevalent defensive clinical practice and poor health literacy among patients, additional challenges hinder DAP implementation [14]. Patients’ lower satisfaction with rural medical services and lower confidence in medical personnel contribute to physicians prescribing antibiotics under patient pressure, leading to overuse. Lastly, while there is unanimous emphasis on coordinated interventions, latent challenges, such as inadequate infrastructure and a shortage of qualified personnel, could hinder the holistic realization of DAP’s potential, requiring comprehensive solutions to overcome these obstacles.

Addressing these factors is vital for the successful adoption and utilization of DAP. Employing the BCW for a thorough analysis allowed us to comprehensively assess the issues at hand and identify potential solutions. In terms of intervention functions and potential solutions, it is imperative to emphasize the importance of equipping both healthcare professionals and patients with comprehensive knowledge. Cultivating a nuanced understanding of DAP, including its advantages, disadvantages, and operational modalities, holds the potential to dispel misconceptions and encourage a constructive orientation toward its integration. Addressing infrastructural challenges and resource limitations is pivotal in facilitating the widespread adoption of DAP.

This includes ensuring that healthcare institutions have appropriate counseling and educational facilities to provide patients with necessary information and support when delaying antibiotic prescriptions, improving patient tracking systems to ensure timely antibiotic prescriptions, and ensuring sufficient technical support for monitoring and reporting the effectiveness of DAP. At the same time, resource limitations must be addressed. This involves training an adequate number of healthcare personnel in the principles and practices of DAP, ensuring ongoing financial support for its implementation, and providing sufficient educational materials and tools to educate healthcare professionals and patients about the importance of DAP. Regarding policy-level interventions, a coordinated advocacy campaign emphasizing the merits of DAP and its crucial role in combating antibiotic resistance takes precedence. The utilization of a diverse range of outreach strategies, spanning both traditional and digital media, holds the potential for far-reaching impact. The varying views on DAP outcomes underscore the need for clear and standardized protocols. These regulatory frameworks can align practices across diverse healthcare settings. Effective oversight by regulatory bodies ensures adherence to established norms and best practices, supported by regular audits, feedback mechanisms, and recognition of exemplary practices as robust regulatory tools.

Although countries such as the UK and Israel have incorporated DAP into their national guidelines to curb unnecessary antibiotic use, its utilization remains limited [15]. Interviews with UK GPs showed mixed feelings about DAP. While some view it as a tool for managing uncertainties, others feel it sends mixed signals about antibiotics’ efficacy [16]. A structural equation modeling model employed in Hubei Province highlighted that physicians’ limited knowledge and disregard of antibiotic resistance contributed significantly to high antibiotic prescribing rates [17]. Therefore, effective DAP adoption requires medical personnel advocacy for patients and increased awareness among healthcare providers regarding antibiotics. Training programs aimed at enhancing antibiotic awareness among physicians and nursing personnel have shown promising results in reducing antibiotic prescribing rates, especially for upper respiratory tract infections in children residing in rural PCIs [18].

The study acknowledges some limitations; notably, the non-inclusion of all domains of the TDF in the interview outline. This may have resulted in some important factors being overlooked. Additionally, potential selection bias in the interviews with adult patients and caregivers should be considered when interpreting the findings, as they may not fully represent the general population.

## 4. Materials and Methods

### 4.1. Study Design

This study utilized a qualitative approach through key informant interviews (KIIs) involving 30 participants selected from 6 county hospitals in Xinjiang Uyghur Autonomous Region. Each KII session was meticulously recorded to ensure accurate documentation of the discussions. The interviews were conducted in accordance with a structured interview outline, as provided in Supplementary Materials, Table S3. To facilitate the interviews, an online meeting software, *VooV* Meeting (version 3.0.0.510, Beijing, China), was employed, allowing for remote communication and efficient data collection.

### 4.2. Setting

The Xinjiang Uyghur Autonomous Region, located in the northwestern part of China, spans an extensive land area of 1,660,000 km^2^ and is inhabited by an estimated population of 25 million, as of 2021. This region holds significance in terms of economic development, as evidenced by its per capita GDP of 9.1 thousand US dollars in the same year, ranking it 18th among the 31 mainland provinces of China. Notably, Xinjiang is distinguished by its expansive rural landscape, comprising 14 prefectures and housing a substantial number of 15,645 primary care institutions (PCIs), thus establishing it as one of the largest rural areas within the nation.

### 4.3. Conceptual Framework

The qualitative data obtained from the interviews were analyzed using an inductive coding approach and categorized using the Theoretical Domains Framework (TDF) [19]. The TDF is an integrative framework that encompasses 33 psychological theories and 128 theoretical constructs, spanning 14 domains related to behavior and behavior change. It offers a theoretical lens to examine the cognitive, affective, social, and environmental influences on behavior, making it widely utilized in implementation science, particularly in health-related psychological and behavioral research. Given its comprehensive coverage of factors influencing behavior, the TDF is well-suited for identifying facilitators and barriers to the potential implementation of DAP. Within the TDF, the COM-B model serves as the core model and consists of three components: capability, opportunity, and motivation. Capability refers to the psychological and physical capacity to engage in a behavior, opportunity represents external factors that can either promote or hinder behavior, and motivation encompasses internal and external factors, such as habits, goals, beliefs, and emotions.

Behavioral change interventions for inappropriate antibiotic use are critical to achieving the milestone of reducing antimicrobial resistance [20]. The Behavior Change Wheel (BCW) is a comprehensive approach that aids in understanding behavior change and enables the design of effective interventions to support it [21]. The BCW consists of three layers, with the COM-B model serving as the central layer. The middle layer consists of nine intervention functions that can be employed to modify behavior, including education, persuasion, incentivization, coercion, training, restriction, environmental restructuring, modeling, and enablement [22]. The outer layer encompasses seven policy categories that can support behavior change, including communication/marketing, guidelines, fiscal measures, regulation, legislation, environmental/social planning, and service provision [22].

**Figure 3:**
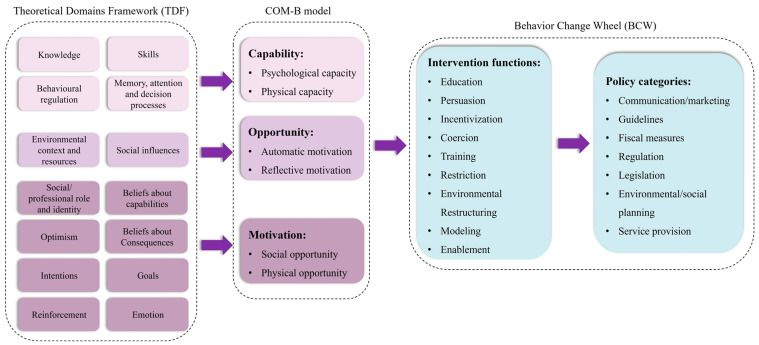
The flowchart based on the Theoretical Domains Framework, Capability, Opportunity, and Motivation model, and Behavior Change Wheel in the study.

In order to establish a practical framework, we developed a linkage between the barriers identified through the TDF and the BCW using the COM-B model, as shown in Figure 3. This integration allowed us to identify suitable intervention functions that can be employed to target the desired behaviors. Furthermore, we identified the pertinent policy categories that can effectively support and strengthen the selected intervention functions. This framework provides a structured approach for designing interventions and implementing policies to promote behavior change in the context of our study.

### 4.4. Recruitment

A two-stage sampling method was employed to select hospitals for qualitative investigation, taking into account economic data and regional availability. In the first stage, regions, prefecture-level cities, and autonomous prefectures were stratified into high-income, middle-income, and low-income groups based on per capita GDP. Per capita GDP data were obtained from official government websites and statistical bureaus of cities in Xinjiang. Two regions were randomly selected from each income group using stratified proportion random sampling.

In the second stage, probability-proportional-to-size sampling was utilized to select healthcare facilities in the sample areas. The selection was based on the number of health facilities in each region, prefecture-level city, and autonomous region. The study team specifically selected six institutions for interviews. Our approach was to ensure balanced representation across various stakeholder groups, including physicians, pharmacists, patients, and caregivers. From the six institutions that were selected, our study team conducted interviews with a specific cohort at each site. In each institution, two physicians (one pediatrician and one non-pediatrician), one pharmacist, one adult patient, and one caregiver of a pediatric patient were interviewed. This amounted to a total of 30 interviewees across all sites. Detailed information on the sampled institutions is listed in Supplementary Materials, Tables S1 and S2.

### 4.5. Interview Outline

The interview outlines for this study were meticulously developed based on the TDF, as detailed in Supplementary Materials Table S3. This framework guided the formulation of comprehensive questions aimed at probing relevant aspects of DAP. To ensure the appropriateness and relevance of the questions, two educators experienced in conducting interviews were consulted during the formulation process. Additionally, a pilot study involving undergraduate students was conducted to assess the clarity and comprehensiveness of the questions. The feedback from the pilot study confirmed that the questions were clear and sufficient for eliciting the desired information.

### 4.6. Data Collection and Analysis

The key informant interviews (KIIs) were conducted in December 2021 in Mandarin to ensure clear and effective communication with the interviewees. Each interview was carried out by a team of two interviewers, with one responsible for conducting the interview and the other for taking detailed notes. Prior to the interviews, the interviewers thoroughly familiarized themselves with the interview content and relevant information to ensure a comprehensive understanding of the topics being discussed. To introduce the research purpose and provide information about the interview process, an interview start-up meeting was held with participants before the interview.

To accommodate the availability of the interviewees, the interviews were scheduled on weekdays and weekends to avoid any interference with their work schedules. Throughout the interviews, audio recordings were made for each KII, and the interviewer concurrently took field notes to capture any additional relevant information or observations. The interviewees were assured of the confidentiality and anonymity of their responses to create a safe environment for open and honest communication.

The original interview recordings were saved in audio format with a designated number and label assigned to each respondent (e.g., N1 + pediatrician + original file of the interview recording) in the .mp4 format. Subsequently, the interviews were transcribed verbatim, with each interviewee assigned a unique number and label (e.g., N1 + pediatrician + original file of the interview recording) and saved in either .doc or .docx format. The content of the interview transcriptions was then organized into a structured document, with each respondent numbered and marked (e.g., N1 + pediatrician + original interview recording file) and saved in either .doc or .docx format.

We analyzed interview data using an inductive coding approach without specialized software, such as ATLAS.ti or NVivo, as detailed in Supplementary Materials Table S4. This decision was driven by the manageable data volume and the specific benefits of a hands-on analysis approach, which allowed for deeper immersion into the data and facilitated a more nuanced understanding and interpretation. The coding process was systematic, involving the derivation of codes from the data, development of themes, and ensuring consistency across the dataset. To ensure coding reliability, we employed cross-checking and validation steps, where multiple team members independently coded portions of the data. Subsequent discussions were held to resolve any discrepancies. For the translation of interview transcriptions into English, initial verification was conducted by two bilingual researchers, proficient in both Mandarin and English. Further accuracy checks were carried out by three additional members of our research group, ensuring that the translations were free from semantic deviations and accurately reflected the original Mandarin responses. This thorough process aimed to ensure the semantic accuracy and faithful representation of the original responses.

## 5. Conclusions

To achieve effective implementation of the DAP, policymakers and healthcare professionals should focus on ensuring the availability of sufficient resources and providing comprehensive education on DAP. By addressing these barriers through resource allocation, education, training, and monitoring, the adoption of DAP can be implemented, providing an opportunity to reduce antibiotic overuse.

## Data Availability

Data are contained within the article and supplementary materials.

## Supplementary Materials

The following supporting information can be downloaded at: https://www.mdpi.com/article/10.3390/antibiotics12121741/s1, Table S1: Stratified sampling scheme of prefecture-level cities in Xinjiang Region; Table S2: The number of stratified samples sampled by the probability-proportional scale in Xinjiang; Table S3: Interview questions for key informant interviews and corresponding theoretical domains; Table S4: Evaluation of implementing the DAP strategy in PHIs based on the Theoretical Domains Framework.
