# Supplementary material for: Exploring Facilitators and Barriers to Delayed Antibiotic Prescribing in Rural Northwest China: A Qualitative Study Using the Theoretical Domains Framework and Behavior Change Wheel
Source: Antibiotics (Basel). 2023 Dec 15;12(12):1741. doi: 10.3390/antibiotics12121741 (PMC10740748; doi:10.3390/antibiotics12121741)
Supplement: Supplementary file 1 [file antibiotics-12-01741-s001.zip › antibiotics-2709460-supplementary.pdf]

antibiotics

MDPI

Supplementary Materials

1. Recruitment

Table S1. Stratified sampling scheme of prefecture-level cities in Xinjiang Region.

Region/Prefecture-Level City/Autonomous Region	GDP per Capita (Million)	Ranking of GDP per Capita	Grouping of Economic Development Level	Number of Prefectures (Percentage)	Number of Selected Settings
Karamay City	21.04	1	High-income group	5 (35.7%)	2
Hami City	9.82	2			
Urumqi City	9.61	3			
Bayingolin Mongolian Autonomous Prefecture	8.94	4			
Changji Hui Autonomous Prefecture	8.26	5			
Bortala Mongol Autonomous Prefecture	7.45	6	Middle-income group	4 (28.6%)	2
Turpan City	6.07	7			
Tacheng Prefecture	5.71	8			
Altay Prefecture	5.16	9			
Aksu Prefecture	4.77	10	Low-income group	5 (35.7%)	2
Ili Kazak Autonomous Prefecture	4.08	11			
Kizilsu Kirghiz Autonomous Prefecture	2.55	12			
Kashgar Prefecture	2.27	13			
Hotan Prefecture	1.49	14			

The data above do not include statistics on gross regional product at the county level under the direct jurisdiction of Xinjiang Autonomous Region. GDP (Gross Domestic Product)

Table S2. The number of stratified samples sampled by the probability-proportional scale in Xinjiang.

GDP per Capita (Million)	Amount of Prefecture-Level City	Selected Amount of Prefecture-Level City	Number of Healthcare Institutions (Percentage)	Sample Size of Medical Institutions
8–22	5	2	4541 (29.0%)	2
5–8	4	2	2670 (17.1%)	1
2–5	5	2	8434 (53.9%)	3
Total	14	6	15,645 (100.00%)	6

GDP (Gross Domestic Product): GDP represents the total monetary value of all finished goods and services produced within a country's borders in a specific time period.

2. Interview questions

Table S3. Interview questions for key informant interviews and corresponding theoretical domains.

Domain	Domain Definition	Interview Questions
Knowledge	An awareness of the existence of something.	Physician: Have you advised patients who are unsure if they need antibiotics during the diagnosis and treatment of respiratory tract infections to avoid taking antibiotics immediately?
		Pharmacists: Have you ever witnessed a doctor refusing to prescribe antibiotics for patients who are uncertain about the necessity of antibiotics during diagnosis and treatment of respiratory infections?
		Adult patients and caregivers: Do you typically take antibiotics for symptoms such as a cough, sore throat, sneezing, or a runny nose?
Skills	An ability or proficiency acquired through practice.	Physician: Do you inquire about the expectations of your patients? And, in the case of a delayed antibiotic prescription, how do you effectively communicate with them?
		Pharmacists: Do patients ask you about the need for antibiotics? How do you respond to patients who are hesitant to take antibiotics? How do you assist such patients?
Memory, attention and decision processes	Retain information, focus selectively, and choose between alternatives.	Physician: How do you determine if a patient is a suitable candidate for implementing a delayed antibiotic prescription?
		Pharmacists: How do you respond to patients who are hesitant to take antibiotics?
		Adult patients and caregivers: How do you obtain antibiotics?
Social/professional role and identity	A coherent set of behaviors and displayed qualities of an individual in a social or work setting.	Adult patients and caregivers: If a doctor made such a suggestion, in addition to communicating with the doctor, do you believe you would benefit from the guidance of a pharmacist or other healthcare professionals to successfully follow the doctor's recommendation?
Beliefs about capabilities	Acceptance of the truth, reality, or validity about an ability, talent, or facility that a person can put to constructive use.	Physician: How many patients do you estimate meet the criteria for "antibiotic avoidance"?
		Pharmacists: How many patients do you think would accept your assistance, and how many would be receptive to the delayed prescription recommendation? Which group of patients is more likely to accept the delayed prescription recommendation?
Optimism	The confidence things happen for the best (includes pessimism).	Physician: In your opinion, would implementing a delayed antibiotic prescription be successful in promoting rational antibiotic use and reducing bacterial resistance?
		Pharmacists: In your opinion, would implementing a delayed antibiotic prescription be successful in promoting rational antibiotic use and reducing bacterial resistance?
		Adult patients and caregivers: If a doctor made such a suggestion, would you accept it? Please explain in detail why or why not.

Beliefs about Consequences	Acceptance of the reality about outcomes of a behavior in a given situation.	Physician: What advantages and concerns do you think there would be if delayed antibiotic prescriptions were implemented in primary hospitals?
		Pharmacists: What advantages and concerns do you think there would be if delayed antibiotic prescriptions were implemented in primary hospitals?
		Adult patients and caregivers: If a doctor made such a suggestion, would you accept it? Please explain in detail why or why not.
Intentions	A conscious decision to perform a behavior or a resolve to act in a certain way.	Physician: Among them, how many would be willing to follow the advice of "delay antibiotics"?
		Pharmacists: As a pharmacist, if a doctor advises a patient not to take antibiotics, would you proactively communicate with the patient to explain the reasons behind the doctor's advice?
		Adult patients and caregivers: Would you go to a hospital to receive a prescription?
Goals	Mental representations of outcomes that an individual wants to achieve.	Physician: How many patients do you estimate meet the criteria for "antibiotic avoidance"?
		Pharmacists: How many patients do you think would accept your assistance, and how many would be receptive to the delayed prescription recommendation?
Reinforcement	Increasing the probability of a response by arranging a dependent relationship, or contingency, between the response and a given stimulus.	Physicians: What policy, system, environmental, or resource incentives do you think are necessary to encourage patients to accept the delayed prescription strategy while ensuring the treatment's effectiveness? Please provide detailed insights.
		Pharmacists: What incentives are needed in policy, system, environment, or resources to enable patients to accept delayed prescribing strategies and reduce antibiotic use while ensuring patient efficacy? Could you please talk about it?
		Adult patients and caregivers: If a doctor made this suggestion, what actions would you expect the doctor to take, and would you be satisfied with their approach?
Emotion	A complex reaction by which an individual attempts to deal with a significant event.	Physicians: What attitudes might patients have toward this recommendation (delayed antibiotic prescription)?
		Pharmacists: What attitudes might patients have toward this recommendation (delayed antibiotic prescription)?
		Adult patients and caregivers: If a doctor made such a suggestion, would you accept it? Please explain in detail why or why not.
Environmental context and resources	Any circumstance of a person's situation or environment that discourages or encourages the	Physicians: What policy, system, environmental, or resource incentives do you think are necessary to encourage patients to accept the delayed prescription strategy while ensuring the treatment's effectiveness? Please provide detailed insights.

	development of skills and abilities, independence, social competence, and adaptive behaviors.	Pharmacists: What policy, system, environmental, or resource incentives do you think are necessary to encourage patients to accept the delayed prescription strategy while ensuring the treatment's effectiveness? Please provide detailed insights.
Social influences	The interpersonal processes that can cause individuals to change their thoughts, feelings, or behaviors.	Physicians: Which group of patients is more likely to accept the prescription delay recommendation?
		Pharmacists: Which group of patients is more likely to accept the prescription delay recommendation?

Table S4. Evaluation of implementing the DAP strategy in PHIs based on the Theoretical Domains Framework.

Domain	Sub-Category	Physician Quote (Exclude Pediatrician)	Pediatrician Quote	Pharmacists Quote	Adult Patient Quote	Caregivers Quote
Knowledge: an awareness of the existence of something						
Facilitators	Knowledge of rational antibiotic use	Antibiotics should not be prescribed when they (patients) are not needed. (N2)	-	Antibacterial drugs are rarely prescribed without a confirmed bacterial infection. (N13)	We often hear about this on the news or read about it online, so try to use antibiotics sparingly. (N19)	-
	Knowledge of symptomatic management	According to the guideline, antibiotics are not recommended for patients with acute suppurative tonsillitis. except for those with a sore throat, fever, and high blood count symptoms. (N12)	Upper respiratory tract infections are caused by viral infections, so antibiotics are unnecessary. Some children do not need medication and will slowly resolve in five to seven days. (N1, N11)	When I practiced in the respiratory department, antibiotics were generally delayed prescribed if a patient had viral pneumonia or bronchial asthma without other infectious symptoms. In addition, in mild cases of COPD, antibiotics are not used at the beginning of treatment. (N23)	-	-
	Knowledge about DAP	-	I had read the research done by the University of Southampton in the UK, and they thought that the DAP would not exacerbate patient's symptoms and	During Antimicrobial Resistance Awareness Week, the National Health Council sent out promotional material this year, and we conducted hospital-wide	-	The doctor also told me to fill the script at home after 2 or 3 days if the symptoms eased. However, if the symptoms did not ease,

			could potentially improve their prognosis and reduce the risk of recurrent disease. (N11)	training on bacterial resistance. (N28)		he would tell me to see a doctor in time. (N30)
Barriers	Worry about aggravation of the condition	There are also risks associated with DAP. For example, the initial viral infection may be combined with a bacterial infection in the later stage for patients with early viral infections. (N22)	-	-	-	-
	Lack of knowledge about DAP	-	-	-	I don't understand these (DAP) either. (N24)	-
Skills: an ability or proficiency acquired through practice						
Facilitators	Good communication skills	Do good missionary work. (N12)	Give him (a patient) a detailed explanation (about why DAP is implemented). (N1)	Explain it to them in plain language. (N13)	-	-
	Be caring	-	We should be a caring person. (N6)	-	-	-
	Be responsible	-	Follow up well and give patients a sense of trust. (N6)	The patient says, "Why didn't the doctor prescribe antibiotic for me? I said I had a sore throat and was uncomfortable, but the doctor didn't prescribe medication for me." In such cases, we will explain to them. (N8)	-	-
	Knowledgeable	It (whether to use an antibiotic) requires our	-	Relatives and friends will consult me by phone, and I will	-	-

		clinical experiences, a deep understanding of respiratory diseases, and a precise grasp of guidelines. (N12)		recommend which medicine is suitable for him. (N8)		
	Using medical knowledge to advocate the correct concept of antibiotic use	We must adopt scientific approaches and theories to explain (to patients) why they are not used. (N12)<br>We will tell him that his blood count, C-reactive protein, and procalcitonin are not high, there is no apparent infection in the lungs, you don't need this antibiotic, and he will definitely be fine. (N22)	I will tell the parents that their child's infection indicators are currently normal and that antibiotics are unnecessary. (N16)<br>I would say, "I want to run a blood routine test for your child to see if there is a combination of bacterial infection. Then we'll see if we need antibiotics." (N21)<br>First, we will explain to the parents that antibiotics must be used cautiously, as misuse can be harmful. Secondly, based on the current symptoms and signs of their child and the results of the laboratory examination, I will explain to her why her child can be temporarily off medication. (N26)	Inform patients that if they use stronger antibiotics, they will develop bacterial resistance and become insensitive to antibiotics, and that fewer antibiotics will be available in the future. (N3)	-	-
Barriers	Hand over the responsibility to the physician	-	-	Communicate with the doctor and let the doctor explain to the patient again. (N3)<br>If they (patient) don't ask me about it (delayed prescribing),	-	-

				we usually don't explain it. (N23)		
Memory, attention, and decision processes: retain information, focus selectively, and choose between alternatives						
Facilitators	According to the clinical symptoms	For asthma, ask if the patient has allergies. If there are some symptoms but no bacterial infection, we conducted delayed prescribing and then decided whether to use it or not after observation. (N17)<br>The patient has no sore throat or fever, no pulmonary rales, and the examination of the image is not consistent with bacterial infection; antibiotics should not be prescribed in this situation. (N2)<br>If he (the patient) has white sputum or dry cough symptoms, correct heart failure first and do not use antibiotics. (N7)	I will conduct a symptomatic treatment and keep the temperature under control. It may be unnecessary if the patient's temperature drops and his mental consequences is quite good. (N11)<br>Pulmonary auscultation and ask for cough symptoms, frequency of cough, and history of seasonal allergies. (N1)<br>If the child is in a better mental state, as he eats and drinks well, his blood count and other auxiliary examination aren't high, proving that the child does not need it (antibiotic). (N6)	-	I usually don't take antibiotics if there is no fever. I take antibiotics if there is a fever and throat discomfort. (N4)<br>If I only have a cough or sneezing and runny nose, I don't take antibiotics immediately; if I have a sore throat, I consider there is inflammation, so I will take antibiotics. (N29)	-
	According to the result of auxiliary examination	We still decide (whether to prescribe an antibiotic or not) by checking the infection indicators. (N22)	The diagnosis is based on the results of the patient's pathogenetic examination. (N26)<br>Check blood count and c-reactive protein for infection indicators. (N21)	-	The doctor gave me a diagnosis according to the results of the examination. (N4)	I will go to the hospital for a blood routine test first, and then I will take it if the doctor tells me to take it or not if he doesn't. (N25)<br>If the symptoms don't relieve, I will go to the hospital for an

					examination and take an antibiotic. (N10)
According to different diseases	It (DAP) depends on the disease. Patients with COPD who have had a good therapeutic effect on previous macrolides may be given antibiotics by the patient's will during the acute episode. (N7)	-	-	-	-
Empirical medication	-	We will administer the medication empirically until the results of his pathogenic tests are available. (N11)	-	(When there is a cough or other discomfort in the throat, sneezing, or runny nose) antibiotics are generally not used. (N14)	-
Hospital management	-	-	From an indicator point of view (antibiotic use), our opinion is not useful (for patients with delayed prescriptions). (N18)	-	-
Follow the physician's decision.	-	-	The physician determines the severity of the patient's symptoms through questioning and thus decides whether to prescribe antibiotics. (N3) Prescribe antibiotics based on the results of the consultation and test indicators. (N18) Antibiotics will be prescribed only according to the patient's treatment effect or specific clinical manifestations. (N23)	If I have symptoms, I'll go to the hospital first. (N24)	Usually, I will take antibiotics on my physician's advice if it is severe. (N15) For example, if my child has an infection, we'll take antibiotics as prescribed by the doctor. (N20)

	Scientific knowledge to support decision-making	-	-	We will tell him that acute upper respiratory tract infection symptoms do not appear immediately, most of which are caused by the virus. We advise him (the patient) to drink more water, rest, and observe more. Antibiotics should be taken after symptoms like fever and infection become apparent. (N8)	-	-
Barriers	Delay in examination	-	Result of pathogenesis examination is often delayed. (N11)	-	-	-
	Empirical medication	-	-	-	I would definitely use cephalosporin in general. Because cephalosporin is for upper respiratory tract infections, we would definitely use it. (N19)	I usually buy my own (antibiotics) at the drugstore for colds like this. (N15)<br>For example, if my child has a high fever and a sore throat, I would consider giving him some (antibiotic). (N5)<br>I will observe for a couple of days and take medication which treats the cold. (N30)
Social/professional role: A coherent set of behaviors and displayed qualities of an individual in a social or work setting. Includes professional identity, professional role, social identity, identity, professional boundaries, professional confidence, group identity, leadership, and organizational commitment.						
Facilitators	Need helps from pharmacists	-	-	-	Pharmacists or other healthcare workers in the hospital should also be helpful. (N9)	I think the pharmacist could also explain to us in more detail (the difference between

						different medicine manufacturers) what we should pay attention to when taking these medicines and whether there are any contraindications. (N30)
	Need helps from physicians	-	-	-	Generally, we would follow the doctor's order. (N19)	We need to communicate more with the doctor. (N20)
	Need helps from nurses	-	-	-	I would listen to the doctor or listen to the nurse. (N14)	After all, nurses are the front-line healthcare workers, so we will have more contact with them. (N15)
Barriers	No extra help needed	-	-	-	I don't think this (help from healthcare workers besides the physician) is necessary. (N4)	I usually don't need to ask any more (questions about delayed prescription) if I follow those instructions to take medicine. (N5)
Beliefs about capabilities: Acceptance of the truth, reality, or validity about an ability, talent, or facility that a person can put to constructive use. Includes self-confidence, perceived competence, self-efficacy, perceived behavioral control, beliefs, self-esteem, empowerment, and professional confidence.						
Facilitators	Trust physicians or pharmacists	Generally, most patients can accept our recommendations. (N2, N7, N12)	Parents can accept it. (N16)<br>Mostly (patients) obedient on the first day. (N21)	Patients are also relatively receptive to the suggestion of DAP after being explained. (N13, N28)<br>As long as the pharmacist proposes, their (patients) compliance is very high. (N8)	I have been seeing this doctor for a long time, so I feel quite trusted in him. I usually take his advice. (N9)<br>After all, doctors must have professional skills, so we must trust them. (N29)	I think the doctor has his expertise, and I will usually listen to his advice. (N5)

Barriers	Not fully trust their doctor or pharmacist	But many patients will ask me to prescribe antiphlogistic medicine. (N27)	The children's grandparents listened to the doctor they knew but didn't listen to us. (N1)<br>Some parents not only listen to the doctor but also listen to the judgment of friends, neighbors, or themselves. (N11)<br>The parent may insist on antibiotics if the child does not get better. (N21)	Most patients will not accept individual recommendations from pharmacists. (N3)<br>If doctors and pharmacists recommend it together, most patients will accept it. (N23)	-	-
Optimism: the confidence things happen for the best (includes pessimism)						
Facilitators	Confidence in the promotion of rational antibiotic use by DAP	Theoretically, DAP should promote rational antibiotic use, and we are very supportive. (N2, N17, N22, N27)	DAP can facilitate the rational application of antibiotics. (N1, N6, N16, N26)	DAP can successfully promote rational antibiotic use and reduce bacterial resistance. (N3, N23, N28)<br>DAP strategy is necessary. (N13)	-	-
Barriers	No confidence in the promotion of rational antibiotic use by DAP	-	-	…it (DAP) doesn't work particularly well either. (N18)	-	-
Beliefs about consequences: acceptance of the reality about outcomes of a behavior in a given situation						
Facilitators	Reduce bacterial resistance	Avoid overuse of antibiotics. (N2)<br>It benefits the standard management of antibiotics and reduces the proportion of antibiotic use and resistance rate. (N7)	It can minimize the side effects of the empirical use of antibiotics and reduce bacteria resistance. (N1)	To a certain extent, it can reduce the problem of medicine misuse and antibiotic abuse. (N28)	-	-
	Reduce costs for patients	-	It can greatly shorten children's hospitalization	It can reduce patient costs. (N3)	-	-

			time, decrease hospitalization costs, and significantly decrease economic pressure. (N1)			
	Reduces risk for patients	It's good for patients. (N7)	There will be less risk for children. (N11)	-	I don't take antibiotics if it's a common cold, and cuz I'm afraid of developing antibiotic resistance. (N4)	What we read on the internet says antibiotic has side effects on the child…something like that… (N25)
	Consolidate physicians' knowledge about antibiotic use	It (DAP) can regulate the behavior of doctors who have recently graduated or haven't graduated for a long time. (N2)	It is helpful for the doctor's cognition, such as giving the pediatrician a clear understanding of antibiotics and the development and occurrence of the disease. (N6)	-	-	-
Barriers	Distrust of consequences	There will be problems if there is no disease-specific diagnosis and one-size-fits-all. (N7)	If the only purpose is to implement the DAP…it will only cause the infection of this child to worsen…Originally, choosing antibiotics like second and third generation can control the infection, but because of the delayed treatment, we will need to select stronger antibiotics. (N1)	It doesn't work very well, either. (N18)	-	-
	Increase hospital stay	Inpatients who do not use antibiotics may prolong their hospital stay and increase the cost burden on patients. (N27)	May lead to a prolonged hospital stay. (N16)	-	-	-

	Patient–doctor disputes	-	There may be patient–doctor disputes over communication. (N6)	-	-	-
	The promotion of the policy is hindered	-	It won't be easy to generalize this strategy, especially in pediatrics. (N26)	This policy is difficult to promote. (N18)	-	-
Intentions: a conscious decision to perform a behavior or a resolve to act in a certain way						
Facilitators	Most patients accept the recommendation	They generally cooperate well with my diagnosis and treatment. (N12)<br>About 70, 80 percent of patients are willing to accept it. (N22)	If parents bring their children to see a doctor, almost 70% to 80% will accept it, which is relatively high. (N1, N6)<br>Parents are generally able to accept a doctor's treatment plan. (N16)	-	-	-
	The doctor or pharmacist is proactive in explaining the reason	-	-	I will proactively explain the risk of antibiotic resistance to patients. (N3)<br>For some patients with certain diseases, we will organize a specialist consultation with the participation of clinical pharmacists. We make further recommendations based on clinical findings and examinations. (N28)	When seeing a doctor, the doctor has explained DAP. (N24, N29)	Yes, after auscultation or some relevant blood routine tests, the doctor would say: "According to the examination, you don't need to take antibiotics, you can take some Chinese patent medicine first." (N30)<br>For example, suppose my two children have a cold and a little cough. In that case, I will take them to the hospital and ask the doctor if they need to take some anti-inflammatory drugs, such as amoxicillin and cephalosporin. The

						doctor will say: "Your child's condition is not very serious, you don't need to take medicine…If you have a bad cough, I would recommend antibiotics or something like that." (N5)
Barriers	Few patients receive advice	Many patients may not accept it. (N27)	Among 50% of the children, only one-third of the parents are willing to accept. (N26)	-	-	-
	The doctor or pharmacist passively explained the reason	-	-	If patients ask, I will explain it to them. If they don't ask me, I usually won't explain. (N23)	-	-
Goals: mental representations of outcomes that an individual wants to achieve						
Facilitators	High proportion of patients were suitable for DAP	Inpatients are about 30–40%; outpatients are about 50–60% or more. (N22) According to the type of disease, less than 30% of heart failure patients need antibiotics immediately. Heart failure caused by chronic obstructive pulmonary disease and pulmonary heart disease may be high, and antibiotics may be required for 70% of the cases. (N7)	At least 50 to 60 percent of outpatients do not need antibiotics. (N16, N21, N26) 70 to 80 percent of children do not need antibiotics. (N6) Maybe half of the patients (can implement DAP). (N26)	Around 60–70% (of patients don't need antibiotics immediately). (N28)	-	-

Barriers	Low proportion of patients were suitable for DAP	If it is a respiratory infection, it is about 10%. (N17)<br>About 20–30% is suitable for not taking antibiotics at first. (N2)	The proportion is not very high. (N11)	About one or two percent a month. (N3)<br>The proportion is relatively low; I think it is 10–20%. (N23)	-	-
Reinforcement: Increasing the probability of a response by arranging a dependent relationship, or contingency, between the response and a given stimulus. Includes rewards, incentives, punishments, consequents, reinforcement, contingencies, and sanctions.						
Facilitators	Standard indicators	-	Statistics of antibiotic usage rate and over-standard rate, as well as microbiology inspection rate. (N1)	Divide the indicators into very fine detail, set specific indicators according to the certain operation of each department, and implement an early-warning mechanism. (N28)	-	-
	Medical personnel training	More training is also needed for doctors. (N12)	Improving the technical level of medical personnel, such as training in antibiotics. (N6)<br>Strengthen pediatric training and policy support. (N6)<br>Strengthen the capacity to train primary doctors in the rational use of antibiotics. (N16)	The physician should have considerable knowledge and experience in antibiotic use.<br>There should be more lectures or resources from higher hospitals. (N23)<br>As clinical pharmacists, we should go deep into clinical departments, avoid using antibiotics without indications, and provide proper and safe knowledge of medicine use for clinical departments. (N13)	-	-
	Reward and punishment system	If there is an unreasonable prescription, a fine of 100 or 200 will be imposed according to the situation.<br>We will lose the opportunity to select excellent and	The proportion of antibiotic use should be linked to performance. (N6, N21)<br>The principles of antibiotic use are strictly following national regulations. (N21)	Include this part of the work in the performance assessment. (N23, N28)	-	-

	advanced doctors every month. (N22) Provide doctors with a certain adaptation period. Suppose the unreasonable use of antibiotics still occurs after the adaptation period. In that case, we can criticize and educate them for the first time, give certain economic punishment for the second time in addition to writing a written review, and then take them to train, specifically to learn and train them on the rational use of antibiotics. If he continues to commit repeated offenses, consider revoking his prescribing rights. For those who do well, we can give praise, as a typical character to publicize, appropriate to a certain amount of economic compensation or reward. (N2)				
Management System	-	There must be a relevant standardized management system to manage, for example, the medical insurance department's intervention and the	Local health institutions should formulate a system for the rational use of antibiotics and strengthen restrictions on the use of antibiotics. (N3)	-	-

			hospital's evaluation mechanism. (N11)			
	Conduct supervision	Strengthen supervision by the unit, the leader in charge, and the medical department. (N12)	Strengthen the supervision by medical administration. (N16)	-	-	-
	Conduct education about antibiotic abuse	Increase education about antibiotic. (N17)	I do feel the need to popularize the risks of antibiotic abuse. (N26)	Increase national awareness. (N3)<br>Doctors, nurses, and pharmacists should educate patients about the pressure of bacterial resistance on society. (N3)<br>Hospitals or healthcare systems should increase awareness of antibiotics. (N23)	-	-
Barriers	Unable to promote	-	-	I don't think any measures will work. (N18)	-	-
Emotions: a complex reaction by which an individual attempts to deal with a significant event						
Facilitators	Accept suggestion	Most patients will accept it. (N2)	The parents' attitudes are positive, and they have high expectations and trust. (N6)	They will not question but trust the pharmacist. (N8)<br>Patients were also relatively receptive to the suggestion of DAP. (N28)	I will accept it (DAP). (N4, N9, N14, N19, N24, N29)<br>After explaining it (DAP) to me in detail, I can accept the opinion. (N4)<br>Just take care of me. (N24)	The acceptance rate is still very high. (N5, N15, N20, N25, N30)
Barriers	Be skeptical	Some people are quite stubborn and may go to the pharmacy to buy medicine by themselves. (N17)	Parents with such aggravation experience may be more cautious or more fearful, afraid that the child	The patient is skeptical. (N3)<br>They want to solve the problem as soon as possible, and they are not very accepting. (N18)	-	I need to hear the doctor's explanation. (N10)

			will be aggravated after returning home, and then they will take more medicine. (N26)<br>Some parents wonder why antibiotics should not be used when the child cannot rule out a bacterial infection. (N11)	They think their children are the best, and they will want to give all the best resources to their children, in which case they may be a little more skeptical. (N23)		
Environmental context and resources: any circumstance of a person's situation or environment that discourages or encourages the development of skills and abilities, independence, social competence, and adaptive behaviors						
Facilitators	National Antibiotic Remediation	The National Health Commission has been regulating antibiotics for 10 years, so we should follow the guidelines for antibiotic use. (N2)	There is now a rational classification of antimicrobial use management. (N21)	The management of antibiotics use is getting stricter. (N8)<br>Special antibiotic regulation activities started in 2011. (N13)<br>Since the COVID-19 pandemic, hospitals have required routine blood tests, so they do not abuse antibiotics. (N3)<br>We have an antimicrobial consultation system with the doctor. (N28)	-	-
Barriers	Insufficient hardware facilities	Bacteriological examinations in primary hospitals cannot be compared with those in higher-level hospitals. (N7)	The inspection report takes a long time, and the inspection accuracy is low. (N1)	-	-	-
	Insufficient human resources	-	There are many patients…It is impossible to communicate the medical history with the patients or tell each child's condition in great detail. (N6)	The workload of pharmacists is getting heavier, and the staff is also tense, so it is impossible to explain one-on-one. (N8)	-	-

	Lack of education	In China, colds and fevers make people nervous.(N17)	Patients have different levels of cognition and acceptance of antibiotics, so they may ask for antibiotics. (N16, N26)	Not enough publicity now. (N23)	-	-
	Poor medical environment	-	Compared with foreign countries, antibiotics in China may be flooded, especially in our primary medical institutions and remote areas. (N11)	The domestic healthcare environment is not good, so patients do not recognize medical institutions. (N23)	-	-
	Insufficient knowledge of doctors	Lack of opportunities for primary physicians to improve their professionalism and learning. (N7)	-	Physicians are less authoritative and trained. (N23)	-	-
	Insufficient regulation	The issue about delaying antibiotics prescription…in fact, may have been quietly done by everyone, but it has not been enforced in words. (N22)	-	Insufficient policy regulation. (N23)	-	-
Social influences: the interpersonal processes that can cause individuals to change their thoughts, feelings, or behaviors						
Facilitators	High educational level	-	Parents with a higher academic level and a wider range of knowledge are easier to accept. (N1, N6, N11, N26)	People with higher education levels are easier to accept. (N3) Patients who know more about antibiotics are also easier to accept. (N23)	-	-
	Parents of less sick children	-	More receptive to parents of children who are rarely sick. (N16)	-	-	-
	Clear knowledge of the disease	-	-	Patients with a clear understanding of their disease	-	-

				or frequent medical practitioners should be more receptive. (N23)		
	Young people	Generally speaking, the youths do what we tell them to do. (N17)	Young parents are more receptive. (N11)	Generally, young adults are easy to accept. (N13)	-	-
Barriers	The elderly	Some older seniors are reluctant to accept. (N22)	-	It's not easy for the elder to accept. (N13)	-	-
	Children who are often sick	-	Parents whose children are often sick and anxious are less receptive. (N26)	-	-	-
	Whether the patient's symptoms are relieved	If the patient's symptoms improve, it is easy to communicate, but if the symptoms do not improve, it is more difficult to communicate. (N7)	-	-	-	-
	Low education level	Some older people with low literacy levels may not understand what you tell them, and sometimes they may not easily accept it when you talk directly like this. (N27)	-	-	-	-
